# Gastrointestinal malignancies at five regional referral hospitals in Uganda

**DOI:** 10.4314/ahs.v17i4.13

**Published:** 2017-12

**Authors:** Siraji Obayo, Luswa Lukwago, Jackson Orem, Ashley L Faulx, Christopher S Probert

**Affiliations:** 1 Mbarara University Teaching Hospital, Mbarara, Uganda and the Uganda Cancer Institute, Kampala, Uganda; 2 Ministry of Health Department of Epidemiology, Kampala Uganda; 3 The Uganda Cancer Institute, Kampala, Uganda; 4 University Hospitals Cleveland Medical Center, Ohio, USA; 6 Institute of Translational Medicine, University of Liverpool United Kingdom

**Keywords:** Gastrointestinal malignancies, Uganda Regional Referral Hospitals, 10-year trend

## Abstract

**Background:**

There is a paucity of published data regarding the trend and distribution of gastrointestinal malignancies in Uganda.

**Objectives:**

To study the trend and distribution of gastrointestinal malignancies over a 10 year period at five regional referral hospitals in Uganda.

**Methods:**

Patient's charts with histologically confirmed diagnoses of gastrointestinal malignancies for the period 2002–2011 were identified. Case information, which included age at diagnosis, sex, and year of diagnosis, primary anatomic site of the tumour and hospitals attended, was retrospectively abstracted. Patient's clinical and demographic features were compared.

**Results:**

Oesophageal cancer was the most common (28.8%) followed by liver (25.8%), stomach (18.4%) and colorectal (14.3%). The mean age at diagnosis for all the cancers was not significantly different in both sexes 54.1, (SD16.1) versus 53.6, (SD 14.7). The highest mean annual number of cases of oesophageal and stomach cancers was 21.8, (SD 15.5) and 16.6, (SD 13.0) respectively from Mbarara Hospital; Lacor had the highest mean annual number of liver cancer cases (21, SD 17.7) followed by Mbale (11.4, SD 8.3). The mean annual number of colorectal cancers was highest in Mbale Hospital (10.3, SD 8.1) followed by Lacor (4.9, SD 3.9). The distribution of oesophageal, liver, stomach and colorectal cancers diagnosed per year across the five referral hospitals was different, P<0.001.

**Conclusion:**

Oesophageal, liver, stomach and colorectal cancer remain the most common gastrointestinal malignancies and their rate is increasing in Uganda. There is a need for awareness, endoscopic and radiological assessment of symptomatic individuals and a need for screening of high index patients.

## Introduction

Accurate national representative data on the trend and distribution of gastrointestinal malignancies in Uganda is sparse, hampering efforts to develop robust public and clinical health interventions.^1^,^2^ The challenges of diagnosis of these malignancies in this resource limited settings is significant because of inadequate endoscopic, radiological facilities and lack of screening programs.^3^–^5^ Recent reports from the Kampala Cancer Registry based in Kyadondo County, which represents about 8% of the Ugandan population, suggest that the trend of gastrointestinal malignancy, specifically oesophageal, stomach, liver and colorectal cancers, is increasing.^6^ However, 90% of the Ugandan population lives in rural areas: they can only access regional referral and district hospitals for their assessment and diagnosis; there is no published data about gastrointestinal cancer cases in these regional referral hospitals. An earlier survey showed variation by geographic location in the distribution of gastrointestinal malignancies, especially for oesophageal and stomach cancer.^7^ Oesophageal cancer was most common around the NorthEast shores of Lake Victoria and rare in Northern and SouthWestern Uganda, while stomach cancer was common in SouthWestern Uganda, but rare in Northern Uganda. Liver cancer was the most frequent cancer in the NorthEast, Northern and SouthWestern Uganda.^7^ Later surveys also found high prevalence of stomach cancer in SouthWestern Uganda.^8^,^9^ Cancer of the large bowel had long been noted to be relatively infrequent in Uganda,^9^,^10^ Data from Kyadondo County shows an increasing trend in the incidence of colorectal cancer.^11^

Attempts to determine the pattern of cancer in Mbarara, SouthWestern Uganda, stomach cancer accounted for 12% of all cancers in males and six percent in females.^12^ The pattern was different from that of Kyadondo County in Central Uganda were stomach cancer was found to be rare, whereas oesophageal was one of the commonest cancer in Kyadondo County but rare in Mbarara.^12^ The pattern of cancer in general and gastrointestinal malignancy in particular is important. National representative data pertaining the pattern and distribution of gastrointestinal malignancies are urgently needed to inform policymakers and support public health messages about cancer to the population where the conditions are most common. Therefore, this retrospective hospital based survey of patients diagnosed with gastrointestinal cancers at five regional-representative hospitals in Uganda was undertaken in order to test the feasibility of obtaining such data from that routinely collected.

## Methods

This was a retrospective study of patients with histological diagnoses of gastrointestinal cancers seen at five referral hospitals in the ten years from January 2002 to December 2011.

**Figure 1:**
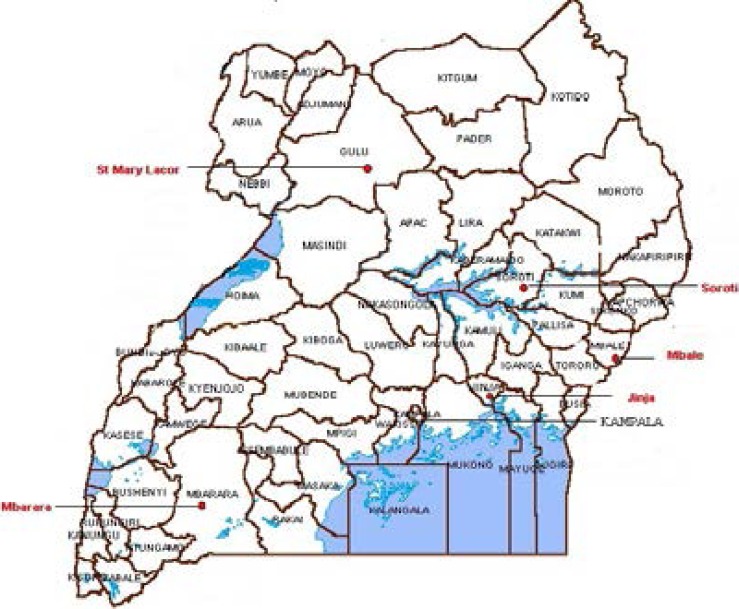
Map showing location of hospitals conducting research

The hospitals were: Mbarara University Teaching Hospital, a 600-bed hospital, one of the three national referral hospitals that serves major part of Western Uganda; St. Mary's Hospital Lacor Gulu, a 483-bed hospital that serves major part of Northern Uganda; Mbale Regional Referral Hospital, a 400-bed hospital and teaching hospital for Busitema University Medical School that serves a population of East and NorthEastern Uganda; Jinja Regional Referral Hospital, a 432-bed hospital in South-Eastern Uganda and a regional referral for Jinja and its neighbouring districts and Soroti Regional Referral Hospital, a 247-bed hospital in NorthEastern Uganda, serving Teso sub-region and its neighbouring districts (Figure 1). The details of patients were retrieved from patients' charts kept in the medical record departments, the surgical wards, operating theaters and histopathology laboratories.

Research assistants compiled case information, including age at diagnosis, sex, and year of diagnosis, primary anatomic site of the tumour, histopathology, and hospital attended using hospital chart abstraction. The information was entered into an epidemiological database for analysis. Analysis was restricted to cases involving the gastrointestinal tract and the study included only regional referral and teaching hospitals where histopathology data would be accessed, thus cases only seen at district hospitals may have been missed and so the completeness of the case series for the regions is uncertain.

The subjects of this study included all patients with histologically confirmed gastrointestinal cancers from the five Referral Hospitals during the period studied. Patients with incomplete data were excluded from the study. The diagnosis of gastrointestinal cancers was performed by endoscopy, double contrast barium meal and laparotomy, and confirmed pathologically by endoscopic and laparotomy biopsies.

## Ethical consideration

We sought permission from individual hospitals prior to accessing charts for data abstraction. The data review was performed as part of cancer survey in collaboration with the Ugandan Ministry of Health with key data collected in aggregate without retaining personal identifying information.

## Statistics

Descriptive analyses of cancers and gender, cases across age groups by gender, cancers across the hospitals, trend and distribution of the cancers with the calendar year were performed in all subjects. The means and their standard deviation of all cancers across the hospitals were calculated. The means were compared across all the five hospitals and p values were computed using a Kruskal Wallis test of means, a p-value of < 0.05 was considered significant using Stata version 12.0

## Results

1468 cases were identified. The average age at diagnosis was 53.9 years (SD 15.6) with a range of 10–98 years and the mean age at diagnosis was not significantly different between males and females (54.1 years (SD 16.1) and 53.6 years (SD14.7) respectively, p = ns). Oesophageal cancer was the most common (28.8%) followed by liver (25.8%), stomach (18.4%) and then colorectal cancer (14.3%), all of which were more common in males (Table 1).

**Table 1:** Anatomical distribution of gastrointestinal cancers reported in 5 hospitals in Uganda between 2002–2011

	Sex			
			
	Male	Female	Frequency	(%)
**Cancer**				

Small intestinal	5	3	8	0.5
Gall bladder	6	6	12	0.8
Biliary	9	5	14	1.0
Pancreatic	39	23	62	4.2
Anal	64	27	91	6.2
Colorectal	110	100	210	14.3
Stomach	162	108	270	18.4
Liver	242	136	378	25.8
Oesophageal	289	134	423	28.8
**Total**	**926**	**542**	**1468**	**100**

**Figure 2:**
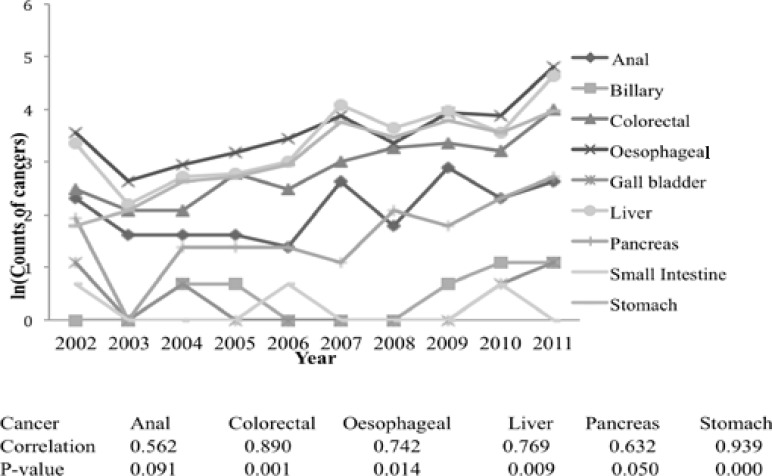
The number of gastrointestinal cancer cases with the calendar year.

There were significantly more cancers in men than women across all age groups. Most cancers occurred in those aged 41–60. The trend and distribution of gastrointestinal malignancies generally increased during the 10-year period with a positive correlation (Figure 2).

Mbarara University Teaching Hospital had the highest mean annual number of cases of oesophageal (22, SD 15.5) and stomach cancers (17, SD 13.0) followed by Lacor. St. Mary's Hospital Lacor had the highest mean annual number of cases of liver cancer (21, SD 17.7) followed by Mbale Regional Referral Hospital (11, SD 8.3). Colorectal cancer was most common in Mbale Regional Referral Hospital (10, SD 8.1) followed by Lacor Hospital (5, SD 3.9). The differences in the mean cases load was significant for oesophageal, liver, stomach and colorectal cancers (P<0.001, Table 2).

**Table 2:** The mean (SD) per year of cancers across the hospitals

	Hospital					
	Jinja	Mbale	Mbarara	Soroti	Lacor	
**Cancer**						**P-value**

Small intestine	0.1 (0.3)	-	0.3 (0.5)	0.2 (0.4)	0.2 (0.4)	0.797
Gall bladder	-	0.3 (0.5)	0.6 (0.7)	-	0.3 (0.5)	0.486
Billary	0.2 (0.4)	-	0.8 (0.6)	0.1 (0.3)	0.3 (0.5)	0.532
Pancreatic	0.3 (0.5)	1 (0.8)	1.7 (1.8)	0.1 (0.3)	3.1 (2.2)	0.768
Anal	0.8 (0.8)	2.5 (2.1)	1.1 (1.0)	1.4 (1.1)	3.3 (2.9)	0.086
Colorectal	1.6 (1.9)	10.3 (8.1)	3.2 (2.6)	1 (0.9)	4.9 (3.9)	<0.001
Stomach	0.1 (0.3)	4.1 (2.5)	16.6 (13.0)	0.3 (0.7)	5.9 (5.2)	<0.001
Liver	0.2 (0.4)	11.4 (8.3)	4.5 (4.2)	0.7 (1.6)	21 (17.7)	<0.001
Oesophageal	1.8 (2.5)	8.4 (4.8)	21.8 (15.5)	1 (1.4)	9.3 (9.2)	<0.001

## Discussion

One thousand four hundred and sixty eight cases of gastrointestinal cancer cases were reviewed, oesophageal cancer was the most common (28.8%). It was most common in Mbarara University Teaching Hospital, Western Uganda with a mean of 22 cases annually. This was similar to earlier studies in Ethiopia, Tanzania and Kenya, which found that oesophageal cancer was the most common gastrointestinal malignancy in those countries.^13^–^16^ The frequency could be due to a number of factors, including an aging population as the life expectancy in Uganda is increasing. Other risk factors are tobacco use, alcohol intake, poor dietary patterns such as consumption of a maize-based diet that is low in fruits and vegetables. Contamination of maize with fungi may lead to the production of fumonisins, which are carcinogenic.^17^ However these modifiable factors are yet to be established in our setting.

Liver cancer was the second most common cancer, (25.7%). This cancer appears to have become more common during the study period. St. Mary's Hospital Lacor in Northern Uganda had the highest mean number of 21 per year. This could be attributable to Hepatitis B infection which is prevalent in this region.^18^,^19^ An earlier study from urban Kampala has shown an increasing trend in liver cancer particularly in women, however the reason for this is unclear.^20^

Stomach (18.4%) and colorectal cancers (14.3%) also showed an increasing trend, similar to studies from Tanzania, Kenya, Ghana and Nigeria.^21^–^25^. The rate of stomach cancer was high with 17 cases per year. On average, in Mbarara University Teaching Hospital, Western Uganda. This could be partly related to diet and partly to precancerous lesions, particularly *Helicobacter pylori* related chronic gastritis that is prevalent there.^26^,^27^

Mbale Regional Referral Hospital had the highest mean number of 10 per year for colon cancer, however we could not find the reason for this in the literature. Anal cancer accounted for 6.2 %. Small intestinal cancers and other malignant tumours of the gastrointestinal tract including pancreatic, biliary and gall bladder cancers were uncommon, as shown in the earlier studies in Ethiopia and Kenya.^13^,^28^,^29^ This could be explained by limited, inadequate facilities and expertise in diagnosing pathology in some of these gastrointestinal tract sites, resulting in under reporting and documentation in these settings.

## Limitations

This study has several limitations. (i) Only regional referral and teaching hospitals where involved, thus cases only seen at district hospitals may have been missed and thus case ascertainment may be incomplete. (ii) We were unable to calculate the age-specific incidence rates and incidence in this study, as there is limited population data. (iii) We cannot exclude diagnostic bias based on interest, expertise and access to diagnostic facilities.

(iv) We only considered histologically-confirmed diagnoses: gastrointestinal cancers like that of pancreas and liver that can be diagnosed with clinical features and imaging only, and those cancers whose histological reviews missed confirmation could have been missed thus under estimating their burden.

## Conclusion

Oesophageal, liver, stomach and colorectal cancer remain the most common gastrointestinal malignancies in Uganda and appear to be increasing. Oesophageal and liver cancers predominate. There is a need for awareness, endoscopic and radiological assessment of symptomatic individuals as well as surveillance of pre-malignant lesions. Screening campaigns have greatly improved the outcomes for patients with colorectal cancer in Europe, stomach cancer in Japan. Vaccination programmes could reduce the transmission of hepatitis B and surveillance of those with cirrhosis used to find cancers.

Treating of *H.pylori* will reduce the prevalence of stomach cancer. Routine fecal screening tests to detect occult blood loss: positive fecal testing should then prompt investigation, however helminthic infections remains more common than colorectal cancer. There is plenty that can and should be done to prevent deaths from gastrointestinal cancers in Uganda.

## Figures and Tables

**Figure 3:**
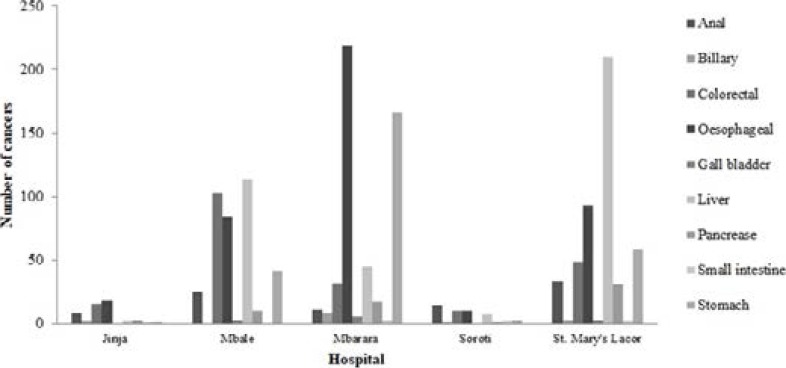
The number of cancer cases across the hospitals
